# LONELINESS AMONG OLDER FAMILY CAREGIVERS: A STUDY OF CAREGIVING INTENSITY, TYPE AND LOCATION BASED ON THE CLSA

**DOI:** 10.1093/geroni/igad104.0620

**Published:** 2023-12-21

**Authors:** Lun Li, Andrew Wister, Yeonjung Lee Lee, Boah Kim

**Affiliations:** MacEwan University, Edmonton, Alberta, Canada; Simon Fraser University, Vancouver, British Columbia, Canada; Chung-Ang University, Seoul, Republic of Korea; Simon Fraser University, Vancouver, British Columbia, Canada

## Abstract

Caring for family members during aging is a risk factor for loneliness among older caregivers (65 years and older). However, loneliness is less understood than other caregiving outcomes (e.g., burden, depression) in caregiving literature. This study aims to examine loneliness among older caregivers using the second wave of data from the Canadian Longitudinal Study on Aging (2015 to 2018). Based on 6603 older caregivers, linear regression was conducted to examine the relationship between loneliness and caregiving intensity, caregiver type and care location, and ANCOVA was performed to examine the intersection of caregiving intensity and caregiver type, as well as caregiving intensity and care location. A higher level of loneliness is significantly associated with spousal caregivers (vs. non-spousal family caregivers), higher caregiving intensity (vs. lower caregiving intensity), and caregiving to someone living in another household or healthcare institution (vs. in the same household). In addition, spousal caregivers with higher caregiving intensity, and older caregivers to someone in healthcare institutions with higher caregiving intensity are two main risk groups for greater loneliness. The findings contribute to a better understanding of the relationship between loneliness and caregiving situations among older caregivers. Remarkably, more service programs are needed to support older caregiver who support loved ones in healthcare institutions.

